# Supplementary material for: Tead1a Initiates Transcriptional Priming Through the TEAD1a/YAP‐Notch1‐Spi1/Cebpα Axis to Promote Neutrophil Fate
Source: Adv Sci (Weinh). 2025 Aug 28;12(41):e05441. doi: 10.1002/advs.202505441 (PMC12591127; doi:10.1002/advs.202505441)
Supplement: Supplementary file 4 — Supplementary Dataset [file ADVS-12-e05441-s004.zip › 3-1.zip!/3-1/01.QC/QCvlnplot_all.pdf]

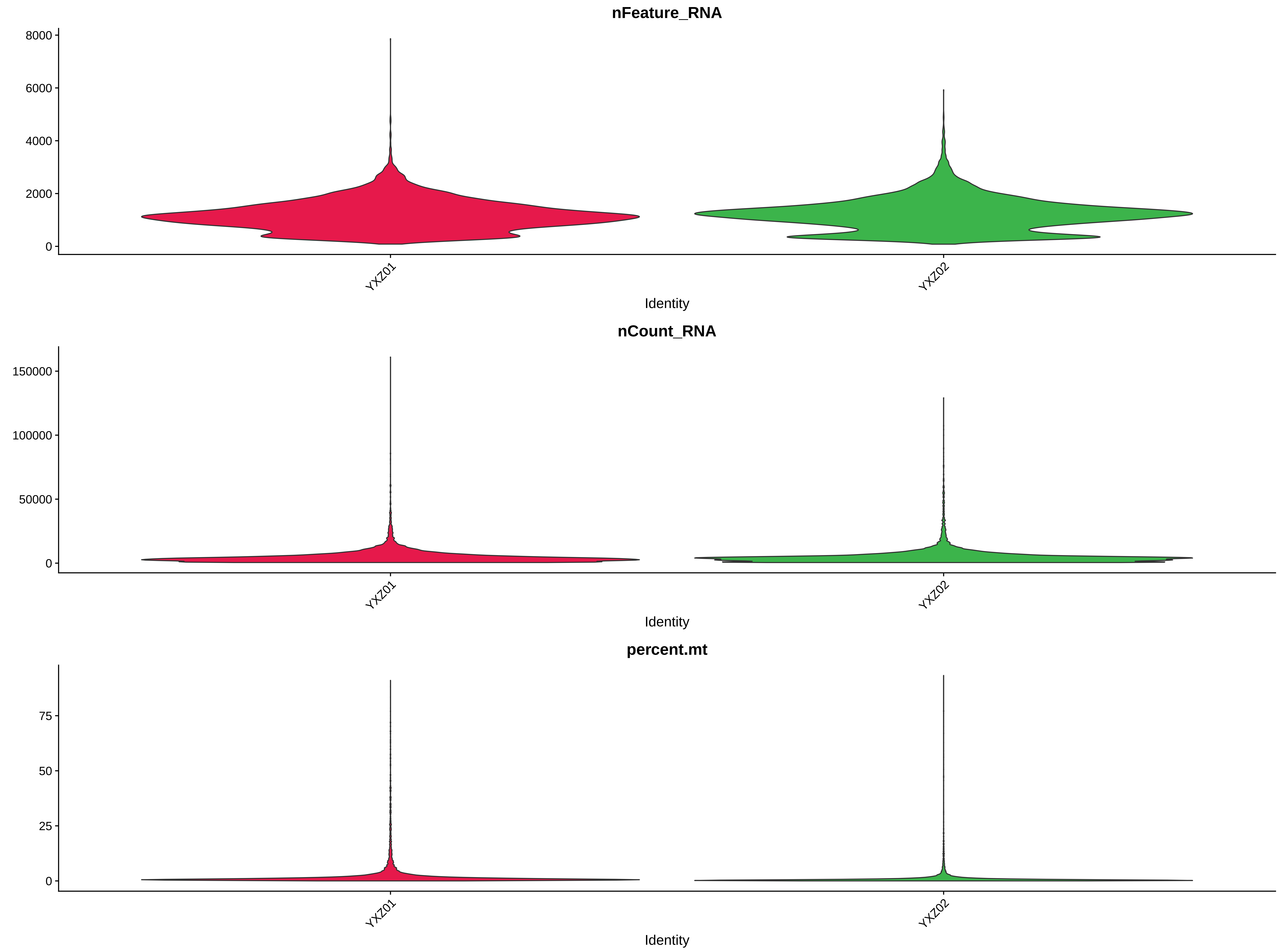

nFeature_RNA
8000
6000
4000
2000
0
YXZ01
YXZ02
Identity
nCount_RNA
150000
100000
50000
0
YXZ01
YXZ02
Identity
percent.mt
75
50
25
0
YXZ01
YXZ02
Identity
